# Aminopeptidase B, a glucagon-processing enzyme: site directed mutagenesis of the Zn^2+^-binding motif and molecular modelling

**DOI:** 10.1186/1471-2091-8-21

**Published:** 2007-10-31

**Authors:** Viet-Laï Pham, Marie-Sandrine Cadel, Cécile Gouzy-Darmon, Chantal Hanquez, Margery C Beinfeld, Pierre Nicolas, Catherine Etchebest, Thierry Foulon

**Affiliations:** 1 Université Pierre et Marie Curie-Paris6, FRE 2852 (CNRS), Protéines : Biochimie Structurale et Fonctionnelle, Structures et Fonctions des Aminopeptidases, Paris, F-75005 France; 2 Department of Pharmacology and Experimental Therapeutics, Tufts University, School of Medicine, Boston, MA 02111, USA; 3 Université Denis Diderot-Paris7, UMR S 726, INSERM, Laboratoire de Bioinformatique Génomique et Moléculaire, Paris, F-75251 France

## Abstract

**Background:**

Aminopeptidase B (Ap-B; EC 3.4.11.6) catalyzes the cleavage of basic residues at the N-terminus of peptides and processes glucagon into miniglucagon. The enzyme exhibits, *in vitro*, a residual ability to hydrolyze leukotriene A_4 _into the pro-inflammatory lipid mediator leukotriene B_4_. The potential bi-functional nature of Ap-B is supported by close structural relationships with LTA_4 _hydrolase (LTA_4_H ; EC 3.3.2.6). A structure-function analysis is necessary for the detailed understanding of the enzymatic mechanisms of Ap-B and to design inhibitors, which could be used to determine the complete *in vivo *functions of the enzyme.

**Results:**

The rat Ap-B cDNA was expressed in *E. coli *and the purified recombinant enzyme was characterized. 18 mutants of the H^325^EXXHX_18_E^348 ^Zn^2+^-binding motif were constructed and expressed. All mutations were found to abolish the aminopeptidase activity. A multiple alignment of 500 sequences of the M1 family of aminopeptidases was performed to identify 3 sub-families of exopeptidases and to build a structural model of Ap-B using the x-ray structure of LTA_4_H as a template. Although the 3D structures of the two enzymes resemble each other, they differ in certain details. The role that a loop, delimiting the active center of Ap-B, plays in discriminating basic substrates, as well as the function of consensus motifs, such as RNP1 and Armadillo domain are discussed. Examination of electrostatic potentials and hydrophobic patches revealed important differences between Ap-B and LTA_4_H and suggests that Ap-B is involved in protein-protein interactions.

**Conclusion:**

Alignment of the primary structures of the M1 family members clearly demonstrates the existence of different sub-families and highlights crucial residues in the enzymatic activity of the whole family. *E. coli *recombinant enzyme and Ap-B structural model constitute powerful tools for investigating the importance and possible roles of these conserved residues in Ap-B, LTA_4_H and M1 aminopeptidase catalytic sites and to gain new insight into their physiological functions. Analysis of Ap-B structural model indicates that several interactions between Ap-B and proteins can occur and suggests that endopeptidases might form a complex with Ap-B during hormone processing.

## Background

Aminopeptidase B (Ap-B; EC 3.4.11.6) activity was originally defined as an exopeptidase able to remove basic amino acid residues from the NH_2_-terminus of peptide substrates [1]. Such activity led to the hypothesis that the enzyme participates in the final steps of precursor processing, namely of neuropeptide and hormone precursors [2-4]. In most cases, proteolytic activation of peptide precursors occurs at arginine and lysine residues and involves the sequential action of an endoprotease and an exopeptidase [3]. Research efforts had focused primarily on endopeptidases cleaving at the COOH-terminus of basic amino acid doublets and on the subsequent participation of a carboxypeptidase E or H (EC 3.4.17.10; reviewed in [5,6]).

However, several endoproteases have been shown to produce cleavage of basic amino acid doublets on their NH_2_-terminal side [7-10]. Moreover, NH_2_-terminally extended form of various peptides, bearing an extra Arg or Lys residue resulting from proteolytic processing of their corresponding precursors, have been described [11-14]. Several recent studies have demonstrated that Ap-B activity occurs in combination with different proteases, such as NRD convertase [15], Cathepsin L [16] and aminopeptidase A [17]. Although clarification of the physiological relevance of Ap-B and identification of all of its *in vivo *substrates is not complete, some of these substrates were recently identified. Indeed, Ap-B processes glucagon into miniglucagon in the α-cells of the islets of Langerhans [15] and could also process angiotensin peptides in rat cardiac fibroblastic cells [17] and enkephalins in various tissues [16]. The enzymatic properties of Ap-B and its expression pattern [4,18-22] lead to the hypothesis that this enzyme could be involved in inflammatory processes [23], development of tumours [24] and Type II diabetes [15].

The biochemical characterization of the purified enzyme isolated from rat testis yielded important information. Ap-B is a monomeric 72 kDa Zn^2+^-dependent exopeptidase, which selectively removes Arg or Lys residues from the NH_2_-terminus of several peptide substrates [4]. In addition to its exopeptidase activity, Ap-B exhibits a residual capacity to hydrolyze leukotriene A_4 _(LTA_4_) into the pro-inflammatory lipid mediator leukotriene B_4 _(LTB_4_), *in vitro *[18]. The bi-functional nature of Ap-B is supported by a close phylogenetic relationship with LTA_4 _hydrolase (LTA_4_H; EC 3.3.2.6; [18]), which hydrolyzes LTA_4 _into LTB_4_, *in vivo*, and exhibits an aminopeptidase activity, *in vitro *[25]. Both enzymes belong to the M1 family of metallopeptidases (consensus Zn^2+^-binding site HEXXHX_18_E; [18,21,25,26]).

The high-resolution crystal structure of LTA_4_H in complex with inhibitors (i.e. bestatin) was recently described [27,28]. This was the first 3D structure determined in the M1 family of metalloexopeptidases to which Ap-B also belongs. Various attempts to crystallize Ap-B have failed due to the low solubility of the enzyme. However, the phylogenetic relationship between LTA_4_H and Ap-B offered the opportunity to model the Ap-B structure using LTA_4_H as a template in order to acquire structural data which will be essential to understand the enzymatic mechanisms, the structure of potential physiological substrates and to design potent inhibitors of Ap-B.

In this paper, we describe the production and purification of the rat Ap-B (rAp-B) in *E. coli*, the characterization of the recombinant protein and its catalytic properties. Eighteen different mutants were generated by site-directed mutagenesis to confirm the functional roles of conserved amino acids belonging to the M1 consensus motif [H^325^EXXHX_18_E^348^], which constitutes the Zn^2+^-binding site. As a step toward the determination of the Ap-B catalytic mechanisms and the structural requirements for the control of substrates specificity, a complete alignment of 500 sequences of the M1 family was performed to point out conserved residues and evolutionary divergences. A part of this alignment was used for the construction of a 3D model of the whole 650 amino acid-long rAp-B based on the crystal structure of human LTA_4_H [27] in complex with Zinc ion and bestatin.

The structural relevance of some previously *in silico *detected consensus sites, such as RNP1 binding motif and Armadillo domain involved in protein-protein interactions is also discussed. Lastly, structural surface differences between Ap-B and LTA_4_H were examined and the results lead to the hypothesis that Ap-B exhibits numerous protein-protein interaction properties according to its putative physiological functions.

## Results

### Expression and purification of the E. coli recombinant rat Ap-B protein

**Figure 1:**
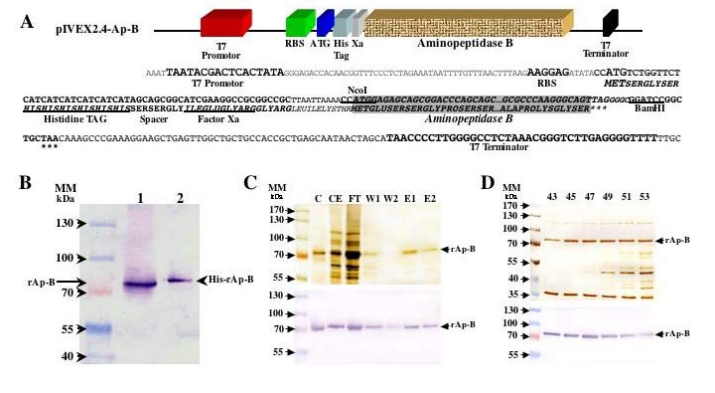
**Cloning of rat Ap-B coding region, expression and purification of the *E. coli *recombinant rat Ap-B protein**. **(A) **Schematic representation of the pIVEX2.4-Ap-B vector. A 1965 bp-long DNA fragment (Aminopeptidase B) containing the rat Ap-B CDS [18] was cloned into the pIVEX2.4 expression vector. T7 promotor, Ribosome Binding Site (RBS), translation initiation codon (ATG), Histidine tag (His Tag), Factor Xa restriction protease cleavage site (Xa or Factor Xa), NcoI and BamHI restriction sites, stop codon (***), and transcription T7 terminator (T7 Terminator) are indicated. **(B) **Western blot analysis of the expression of the recombinant rAp-B in *E. coli*. Lane 1 contains a purified recombinant rAp-B produced in a baculovirus expression system ([29]). Lane 2 contains a crude protein extract from pIVEX2.4-Ap-B transformed cells. Each sample (2 μL) was diluted in 20 μL of 1× Laemli buffer to avoid buffer effects during electrophoresis. Arrows indicate immunoreactive proteins corresponding to the baculovirus produced rAp-B (rAp-B; 650 amino acid residues; [29]) and the *E. coli *expressed rAp-B (His-rAp-B; 673 amino acid residues), respectively. **(C) **Purification using Ni-NTA column. The different protein extracts analyzed are the following: C, rAp-B purified from baculovirus expression system used as a positive control; CE, crude protein extract from pIVEX2.4-Ap-B transformed BLi5 cells (5 μL); FT, proteins released after passage through the Ni-NTA column (20 μL); W1 and W2, proteins released after the first and second washing of the Ni-NTA column, respectively (20 μL); E1 and E2, proteins collected after the first and second elution step, respectively (20 μL). **(D) **Purification of recombinant rat Ap-B using DEAE ion exchange column. Different fractions (43, 45, 47, 49, 51, 53) collected from the ion exchange column and containing rAp-B activity were analysed (20 μL per fraction). **(C, D) **Samples were run on SDS-PAGE and either stained with silver salts (upper panel) or transferred to a nitrocellulose membrane for western blotting (lower panel). Arrows indicate the *E. coli *expressed rAp-B (rAp-B). **(B, C, D) **Relative molecular masses are indicated in kilo Daltons (MM, kDa).

A 1965 bp-long DNA fragment containing the rat Ap-B CDS was cloned into the pIVEX2.4 expression vector (Fig. 1-A). Then, the recombinant rat Ap-B protein (rAp-B) was produced with this T7 promoter-driven plasmid in a BLi5 *E. coli *strain. The translated product comprises a histidine tag (His-tag) at the NH_2_-terminus followed by a factor Xa restriction protease cleavage site and the rAp-B sequence, providing a 673 amino acid protein (His-rAp-B; Fig. 1-A, -B). The difference in length due to the fused His-tag/Factor Xa site tail (673 versus 650 amino acid residues) leads to a slight difference of electrophoretic mobility, which is observed in Western blot analysis (Fig. 1-B). This latter also shows that His-rAp-B is properly expressed in this prokaryotic expression system.

Cloning of rAp-B as a NH_2_-polyhistidine-tailed protein allows a rapid purification procedure. Figure 1-C shows that there is a significant loss of desired His-rAp-B during the first washing. Any attempts to increase the binding specificity of the histidine tag during this step led to a decrease of specificity during the elution steps. Gel electrophoresis of the elution fractions E1 and E2 (1.5 mL each) showed only one band with a relative molecular mass around 73 kDa (Fig. 1-C). Both fractions were pooled and concentrated to 1 mL in Tris-HCl 50 mM pH 7.2. The final concentration of the purified His-tag rAp-B was about 160 μg.mL^-1^. Consequently, an adequate quantity of enzyme is obtained with this purification procedure since we obtained 160 μg of purified protein from 50 mL of cell culture corresponding to a purification rate of 3.2 mg.L^-1^.

In order to identify potential differences in the kinetic parameters of the enzyme activity due to the purification procedures, the His-tagged rAp-B was also semi-purified using a DEAE Trisacryl Plus M ion exchange column eluted with 80 mM KCl (Fig. 1-D). Gel electrophoresis of the elution fractions containing Ap-B activity (5 mL each) showed several bands with a relative molecular mass between 35 to 115 kDa (Fig. 1-D). Fractions 43 to 47, which contain 4 main detectable bands on the silver stained SDS-PAGE were pooled (25 mL; Fig. 1-D) and concentrated to 5 mL in Tris-HCl 50 mM pH 7.2. The final concentration of the semi-purified proteins was ~40 μg.mL^-1 ^*versus *80 μg.mL^-1 ^for fractions 48 to 53. Within the limits of a visual quantification, this analysis shows that the amounts of His-tagged rAp-B corresponds to about 30% of the total proteins leading to an estimated purification rate of about 1 mg.L^-1^. Note that subsequent exclusion chromatography steps do not allow the separation of the contaminant proteins despite their molecular mass differences (data not shown).

A Western blot analysis completes the identification of the proteins resulting from these purification procedures (Fig. 1-C, -D, lower panels) and shows that no degradation products were generated.

### Characterization of the purified His-tagged rat Ap-B activity

Kinetic constants for the enzymatic reaction of His-rAp-B with L-Arg and L-Lys β-NA were estimated from Lineweaver-Burk plots of 1/v against 1/[S] and Hanes-Woolf plots of [S]/v against [S] in the substrate concentration range 1–35 μM (Table 1).

**Table 1:** His-rAp-B kinetic parameters for the hydrolysis of L-Arg β-NA and L-Lys β-NA.

**Substrate**	**Enzyme His-rAp-B (nM) *(Ni-NTA purification)***	**Km (μM)**	**Vmax (nM.s^-1^)**	**kcat (s^-1^)**	**kcat/Km (M^-1^.s^-1^)**
L-Arg-β-NA	4,66	108 ± 13	146 ± 17	20 ± 3	1.85 × 10^5^
L-Lys-β-NA	21.9	135 ± 30	65 ± 7	3 ± 0.35 s^-1^	2.2 ± 0.2 × 10^4^
**Substrate**	**Enzyme His-rAp-B (nM) *(DEAE semi-purification)***	**Km (μM)**	**Vmax (nM.s^-1^)**		
L-Arg-β-NA		137 ± 0.11	200 ± 0.18		
L-Lys-β-NA		170 ± 0.38	90 ± 0.18		
**Substrate**	**Enzyme His-rAp-B (nM) *(from insect cells)***	**Km (μM)**	**Vmax (nM.s^-1^)**	**kcat (s^-1^)**	**kcat/Km (M^-1^.s^-1^)**
L-Arg-β-NA	14	65	295	21	3.2 × 10^5^
**Substrate**	**Enzyme rAp-B (nM) *(DEAE semi-purification from rat testes)***	**Km (μM)**	**Vmax (nM.s^-1^)**		
L-Arg-β-NA		20	60		
L-Lys-β-NA		36	42		

Km, Vm and kcat values are the means ± standard error of three independent series of tests in which each point was performed in triplicate. Comparative values, obtained with His-r-Ap-B purified from baculovirus infected insect cells [29] and rat testes Ap-B [4], are indicated.

The enzymatic activity of His-rAp-B is probably higher than estimated above. Indeed, a decrease of activity due to the higher purification of the enzyme was previously described [4,29]. Meanwhile, this purification step is required to calculate the kinetic constant such as kcat and consequently kcat/Km. Equivalent measurements using the DEAE semi-purified His-rAp-B show that enzyme activity is similar to that of Ni-NTA (Table 1). Consequently, the semi-purified enzyme can also be used in activity assays. The comparison of kinetic values between baculovirus recombinant Ap-B [29], rat testes enzyme [4] and *E. coli *recombinant protein shows that this latter constitutes a reliable tool to study the enzymatic mechanism of Ap-B, avoiding long procedures of purification (Table 1).

**Figure 2:**
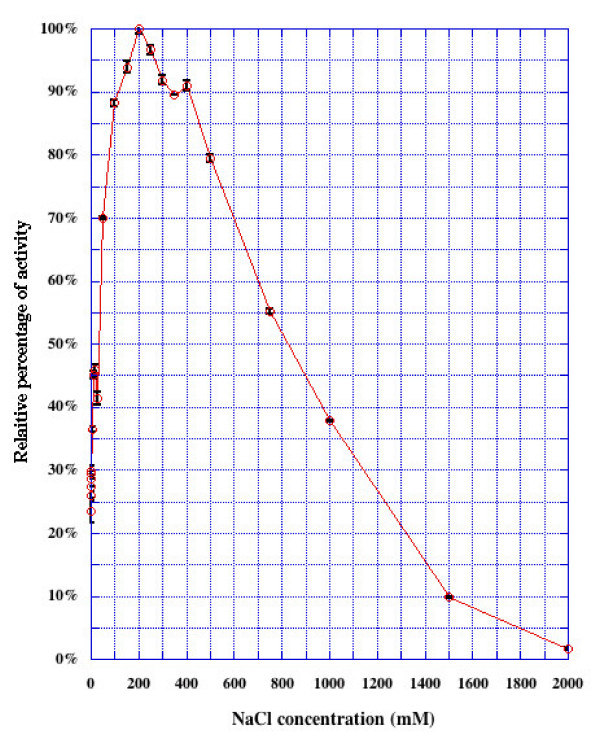
**Effect of NaCl concentration on His-rAp-B enzymatic activity**. Activity was measured using the standard assay method described in Materials and Methods with the indicated NaCl concentrations. Activities are expressed relative to the maximum activity (100%) of the His-rAp-B enzyme obtained in presence of 200 mM NaCl. Bars represent SEM of 3 or more separate assays.

Addition of 0.2 M NaCl with either the purified rat testis Ap-B protein or the recombinant enzyme expressed in baculovirus system, results in a 3-time increase in activity [4,29]. Consequently, the effect of NaCl was studied by assaying bacterial recombinant His-rAp-B activity in the presence of different concentrations of NaCl ranging from 0 to 2 M in 0.1 M borate buffer pH 7.4 (Fig. 2). A similar increase of activity (about 3 fold) is observed in the range 75–400 mM NaCl with a maximum increase at 200 mM, followed by a strong inhibition.

The inhibitor profile of His-rAp-B was studied using the L-Arg-β-NA as substrate. As shown in Table 2, the recombinant enzyme shows metallopeptidase properties with an inhibition of its activity with EDTA and O-phenanthroline in the milli- and micromolar concentrations, respectively. The rAp-B activity is not affected by PMSF, a serine protease inhibitor. As expected, aminopeptidase inhibitors such as bestatine (Ki = 50 nM *versus *100 nM for Ap-B purified from insect cells) or arphamenine A and B (Ki = 20 nM *versus *40 nM for Ap-B purified from insect cells) work in micromolar concentrations. Therefore, the recombinant enzyme exhibits the similar enzymatic properties to the testis protein.

**Table 2:** Effect of inhibitors on His-rAp-B activity.

		**Percentage of inhibition *(L-Arg-β-NA Substrate)***	
		
**Inhibitors**	**Final concentration**	Testis Enzyme*	***E. coli *Enzyme**
**O-phenanthroline**	500 μM	76	80
	250 μM	55	67
	100 μM	27	6
	50 μM	-	0
			
**EDTA**	10 mM	95	71
	5 mM	84	65
	1 mM	34	53
			
**NEM**	1 mM	45	71
			
**PMSF**	500 μM	0	5
			
**Bestatin**	100 μM	-	100
	50 μM	100	98
	1 μM	68	94
			
**Arphamenine A**	1 μM	100	100
	500 nM	-	97
	200 nM	-	92
	100 nM	-	87
			
**Arphamenine B**	1 μM	100	100
	500 nM	-	100
	200 nM	-	99
	100 nM	-	95

The percentage of inhibition was calculated by taking as reference the amount of substrate conversion by His-rAp-B in absence of inhibitor (see Materials and Methods section). Values are the average of three independent series of tests in which each point was performed in triplicate. (* from [4])

### Site-directed mutagenesis of the HEXXHX_18_E rAp-B motif

In order to probe the functional roles of several amino acids previously identified in other members of the M1 family (For review, see in [30]), an investigation of the conserved HEXXHX_18_E motif was conducted by site-directed mutagenesis (Table 3). Mutants were expressed in BLi5 *E. coli *strain. The corresponding proteins were then purified or semi-purified either by Ni-NTA agarose chromatography or DEAE ion exchange column, respectively and showed a single band on SDS-PAGE and Western blot, similar to wild type enzyme (data not shown). Conservative mutants such as H325H, E326E, H329H and E348E constituted positive controls.

**Table 3:** Activity of the wild type and mutant enzymes.

**Mutation site**	**Number of mutants**	**Mutated codon**	**Mutants**	**Percentage of activity**
H_325_		CAC	Wild type	100
	3	CAT	H	100
	2	TAT	Y	0
	5	GCC	A	0
	6	TTC	F	0
E_326_		GAG	Wild type	100
	2	GAA	E	100
	2	GCG	A	0
	4	CAA	Q	0
	3	GAT	D	0
	7	CAT	H	0
H_329_		CAC	Wild type	100
	3	CAT	H	100
	4	GCC	A	0
	3	TAT	Y	0
	4	TTC	F	0
E_348_		GAA	Wild type	100
	8	GAG	E	100
	1	CAG	Q	0
	2	GAC	D	0
	4	GCG	A	0
	3	CAC	H	0

The number of mutants analysed, the mutated codon and its corresponding amino acid, as well as the percentages of enzyme activity are indicated. The position in the rat Ap-B sequence of the targeted amino acid is numbered. The percentage of activity was determined using L-Arg β-NA as substrate. Activity assays were performed in triplicate with enzymes collected from both purification methods.

Mutagenesis of any of the three zinc binding ligands of the HEXXHX_18_E conserved motif (H^325^, H^329^, E^348 ^in rat Ap-B sequence or of the critical general base of the peptidase reaction E^326 ^led to complete loss of aminopeptidase activity (Table 3). As shown by E326Q/D and E348Q/D mutants, the negative charge and the length of the side chain seem to be important for the glutamate residue functions. On the other hand, results obtained with the H325Y/F and H329Y/F mutants suggest that nitrogen atoms of histidine play a role in chelating the Zn^2+ ^cation.

### Multiple sequence alignment

In order to establish the basis for molecular modelling and further site-directed mutagenesis studies, we performed a multiple sequence alignment of proteins belonging to the M1 family of aminopeptidases. A BLASTP search of the Uniprot database using rat Ap-B sequence retrieved 512 protein sequences and 500 of them were initially selected to perform a CLUSTALW alignment. Redundancy inside the selected sequences was conserved to identify partial sequences (NH_2_- and/or COOH-terminal truncations; internal deletions), sequence insertions and sequencing errors. These latter sequences were deleted from the multiple alignment. Consequently, 403 proteins varying in length from 407 (Q6L8I5, *Kitasatospora setae*) to 1890 (Q22531, *Caenorhabditis elegans*) amino acid residues were aligned.

Examination of the longest sequences (Q7PYD3, 1800 amino acids, *Anopheles gambiae*; Q61J79, 1875 amino acids, *Caenorhabditis briggsae*; Q22531, 1890 amino acids, *Caenorhabditis elegans*) showed that these proteins are translated from a duplicated gene encoding in tandem two similar aminopeptidases of the M1 family. The COOH-terminal domains encompassing the second aminopeptidase module of these 3 proteins were deleted from the alignment. Subsequent alignments of these domains with the consensus signature exhibited identical conserved amino acid residues (data not shown).

**Figure 3:**
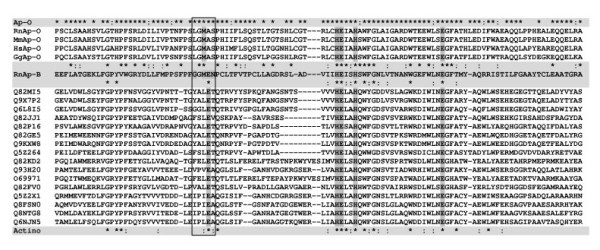
**Partial multiple alignment of aminopeptidase O, rat aminopeptidase B and Actinobacteria aminopeptidase sequences**. Sequences were aligned with ClustalW using the Blosum62 matrix. Multiple alignment of the central Zn^2+ ^chelating region of Ap-O from Rattus norvegicus (RnAp-O; AMPO_RAT), Mus musculus (MmAp-O; AMPO_MOUSE), Homo sapiens (HsAp-O; Q5T9B3), Gallus gallus (GgAp-O; XP_425041.1) and aminopeptidases from Actinobacteria [Streptomyces avermitilis (Q82MI5, Q82JJ1, Q82P16, Q82GE5, Q82KD2, Q93H20, Q82FV0); Streptomyces coelicolor (Q9X7P2, Q9KXW8, O69971); Kitasatospora setae (Q6L8I5); Nocardia farcinica (Q5Z264, Q5Z2X1); Corynebacterium efficiens (Q8FSN0); Corynebacterium glutamicum (Q8NTG8); Corynebacterium diphtheriae (Q6NJN5)] is shown. The corresponding part of the Rattus norvegicus Ap-B sequence (RnAp-B; AMPB_RAT) is indicated, as well as the conserved amino acid residues between Ap-B and Ap-O (above RnAp-B sequence) and between Actinobacteria aminopeptidase sequences (below RnAp-B sequence). Conserved amino acid residues between Ap-O proteins or between Actinobacteria proteins are shown above (Ap-O line) and below (Actino line) the multiple alignment, respectively. In the consensus lines, amino acids invariant in all sequences are indicated by stars (H) and colons (:) indicate similar residues according to the following rule: T:A:S; I:L:V:M; K:R:H; D:E:Q:N; F:Y; G; P; W; C. The location of the GXMEN motif is boxed and the conserved amino acid residues are highlighted in the corresponding Ap-O and Actinobacteria sequences. The HEXXHX_18_E motif is also highlighted. Dashes typify gaps.

On the other hand, analysis of the shortest sequences revealed interesting data. Indeed, these proteins display only one of both consensus motifs of the M1 family, the Zn^2+^-binding site HEXXHX_18_E. The second consensus motif GXMEN that is classically located about 22 to 38 amino acids upstream the Zn^2+^-binding motif is missing. The glutamate alone seems to be conserved (Fig. 3).

These proteins are encoded by the genomes of *Actinobacteria *(*Streptomyces avermitilis*: Q82MI5, 497 amino acids; Q82JJ1, 463 amino acids; Q82P16, 454 amino acids; Q82GE5, 469 amino acids; Q82KD2, 489 amino acids; Q93H20, 483 amino acids; Q82FV0, 532 amino acids; *Streptomyces coelicolor: *Q9X7P2, 506 amino acids; Q9KXW8, 473 amino acids; O69971, 512 amino acids; *Corynebacterium diphtheriae: *Q6NJN5, 450 amino acids; *Corynebacterium efficiens: *Q8FSN0, 466 amino acids; *Corynebacterium glutamicum: *Q8NTG8, Q6M822, 460 amino acids; *Nocardia farcinica*: Q5Z264, 493 amino acids; Q5Z2X1, 461 amino acids; *Kitasatospora setae*: Q6L8I5, 407 amino acids; *Deinococcus radiodurans*: Q9RVZ5, 472 amino acids). These 18 closely related enzymes clearly constitute a sub-group in the M1 aminopeptidase family (Fig. 3).

The recent publication of the sequence of human aminopeptidase O (Ap-O; [31]), an aminopeptidase related to Ap-B and LTA_4_H, led us to compare this sequence to that of other members of the M1 family. Note that the Uniprot database only contains the human Ap-O sequence (Q5T9B3, Q8WUL6, Q96M23). Although Ap-O is most closely related to Ap-B, the global identity between both enzymes is very low (23.5%; LTA_4_H *versus *Ap-O, about 20 %). Due to a large discrepancy in length (Ap-B, 650 amino acids; Ap-O, 819 amino acids) and weak percentages of identity and similarity (32.8%), the evolutionary relationships between these two proteins remains unclear. In addition, analysis of the M1 consensus signature [GXMENX_22–38_HEXXHX_18_E] shows that the GXMEN motif is lacking, except the Met residue in the Ap-O human sequence (Fig. 3). This observation was confirmed by analysis of different sequences extracted from the nr database (Genbank CDS translations + RefSeq Proteins + PDB + Swissprot + PIR + PRF) such as AMPO_RAT (*Rattus norvegicus*), AMPO_MOUSE (*Mus musculus*), XP_425041.1 (*Gallus gallus*), XP_541256.1 (*Canis familiaris*) and CAG10896.1 (*Tetraodon nigroviridis*) (data not shown). In the M1 family, Ap-O constitutes the unique member of a second emerging sub-group (Fig. 3).

**Figure 4:**
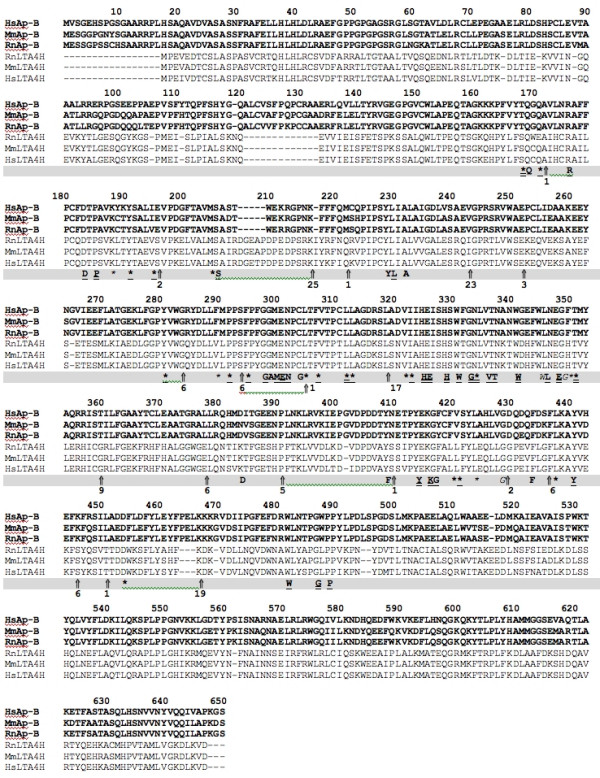
**Multiple alignments of mammalian aminopeptidase B and leukotriene A_4 _hydrolase sequences and M1 family signature**. Sequences were aligned with ClustalW using the Blosum62 matrix. The multiple alignment of Ap-B (in bold-face) from Rattus norvegicus (RnAp-B; AMPB_RAT), Mus musculus (MmAp-B; NP_663392.1), Homo sapiens (HsAp-B; CAC14047.1) and LTA_4_H from Rattus norvegicus (RnLTA4H; S20444), Mus musculus (MmLTA4H; JN0066), Homo sapiens (HsLTA4H; NP_000886.1) is shown. Gaps are indicated by dashes. Conserved amino acid positions emanating from the whole M1 family multiple alignments (382 proteins) are shown below the alignment. In this consensus line, amino acids invariant in more than 98% of the sequences (> 377/382) are in bold-face and double underlined, residues conserved in more than 90% of the primary structures (> 343/382) are in bold-face and underlined, those conserved in more than 85% of the sequences (> 324/382) are in bold-face and amino acids conserved in more than 80% of the protein sequences (> 305/382) are shown in normal type. Stars indicate similar residues according to the following rule: T:A:S; I:L:V:M; K:R:H; D:E:Q:N; F:Y; G; P; W; C and are typified according to the percentages described above. Vertical arrows point out the presence of one or several gaps (see the number below or alongside the arrows) just after the indicated amino acid residue in the multiple alignments of 382 sequences.

The 382 remaining sequences, including Ap-B and LTA_4_H proteins, exhibit both consensus motifs of the M1 family and thus constitute a third sub-group in the latter. The multiple alignment of these 382 sequences, varying in length from 611 (leukotriene A_4 _hydrolases) to 1025 (Thyrotropin-Releasing Hormone degrading enzymes) amino acids, permitted the identification of a prominent conserved region of approximately 460 residues (including gaps) containing the GXMEN motif and the Zn^2+^-binding site (HEXXHX_18_E; Fig. 4). This region overlaps only 316 amino acids residues in the Ap-B sequences (from Thr_168 _to Pro_389_; Fig. 4). This alignment reveals 61 highly conserved positions. Their conservation among several divergent prokaryote and eukaryote peptidases points their potential importance in maintaining the structural integrity of the proteins or in directly interacting with the substrates.

A subsequent search of the Uniprot database with the PATTERN program for consensus amino acid signatures using conserved residues highlighted in the multiple alignment revealed that the HEXXHX_3_G [DENQ]X_6_WX_6_E motif was specific to the M1 family (469 proteins detected). The EX_15–45_HEXXHX_3_G [DENQ]X_6_WX_6_E signature detected specifically all the M1 proteins *minus *the Ap-O sequences. The conception of different signatures using other conserved amino acids such as in region 168–210 or 409–441 failed.

### Molecular modelling of Ap-B

Sequence alignments indicated that rat Ap-B shares 33% identity and 48% similarity with LTA_4_H. LTA_4_H and Ap-B (611 and 650 amino acids respectively) exhibit similar sizes. Consequently, the crystal structure of LTA_4_H constituted a suitable template to model the Ap-B 3D structure (see Additional File 1). The overall structure can be described as follows: Ap-B protein is folded into three different NH_2_-terminal (residues 1–235), catalytic (residues 236–484) and COOH-terminal (residues 485–650) domains packed in a triangular arrangement with dimensions of about 78 × 55 × 51 Å *versus *about 85 × 65 × 50 Å for LTA_4_H. The catalytic centre is located along a deep cleft, created between the three domains. The NH_2_-terminal domain is composed of a large seven-stranded mixed β-sheet surrounded by two smaller β-sheets. This NH_2_-terminal domain seems to shape an envelope, such as in LTA_4_H, presenting a large concave surface to the solvent.

**Figure 5:**
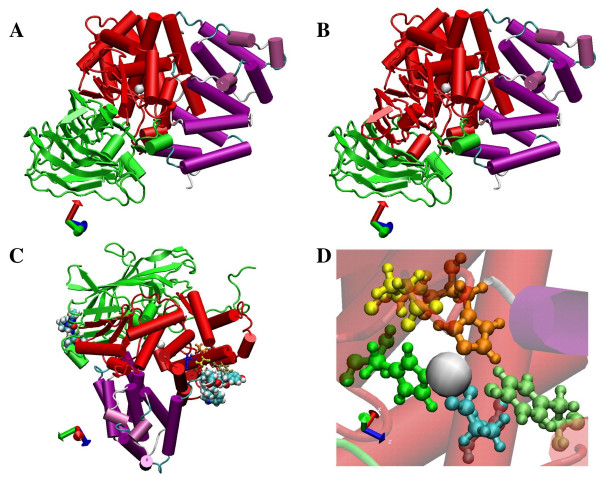
**Model of the Ap-B structure, consensus motifs and Zn^2+ ^close residues**. **(A) **Domain structure of rat Ap-B according to [27]. The NH_2_-terminal, catalytic and COOH-terminal domains are colored in green, red and purple, respectively. The Zn^2+ ^ion is depicted as a sphere and colored in grey. **(B) **Representation of the domain structure of rat Ap-B supported by multiple sequence alignments and conserved amino acid residues using the same colors than above. **(C) **Representation of the Proline-rich loops (position 47–57 and 485–499; depicted as space fill) and of the putative RNP1 consensus motif (position 416–423; depicted as ball-and-stick) on the whole Ap-B structural model. **(D) **Magnification of the catalytic centre showing the high proximity between the Zn^2+ ^ion (grey sphere) and, on one hand, the three Zn^2+ ^ligands (His^324^, orange ; His^329^, green ; Glu^348^, blue) and, on the other hand, the catalytic residue Glu^325 ^(yellow) and the putative proton donor in the catalytic reaction (Tyr^413^, light green). The remaining part of the structure is depicted as transparent material. The figure was created using the molecular graphics software Visual Molecular Dynamics (VMD 1.8.2; [60].

The structures of the Ap-B and LTA_4_H catalytic domains are very similar. This domain consists of two lobes, one mainly α-helical and one mixed α/β (Fig. 5-A). The Zn^2+^-binding site (H^325^EXXHX_18_E^348^) in Ap-B model remains between the two lobes, such as LTA_4_H [27].

The Ap-B Zn^2+ ^ion is coordinated by His^325^, His^329 ^and Glu^348 ^residues of the H^325^EXXHX_18_E^348 ^motif (Fig 5-D). The distances between the Zn^2+ ^ion and His^325^, His^328 ^and Glu^348 ^are 2.14 Å, 2.1 Å and 1.98 Å, respectively. A more precise definition of the structure of the catalytic domain could be done thanks to the contribution of sequences alignments. Indeed, multiple alignments highlight a 316 conserved amino acids region that lead to the hypothesis of a functional role in the exopeptidase activity of this entire region. In this case, the catalytic domain of LTA_4_H and Ap-B proteins extend into the NH_2_-terminus structural domain previously described ([27]; residues 168–489 for Ap-B) adopting a horseshoe shape (Fig. 5-B). As expected, due to the high percentage of similarity between both proteins in this region, the hydrophobic cavity constituting the putative substrate-binding pocket is conserved (data not shown).

**Figure 6:**
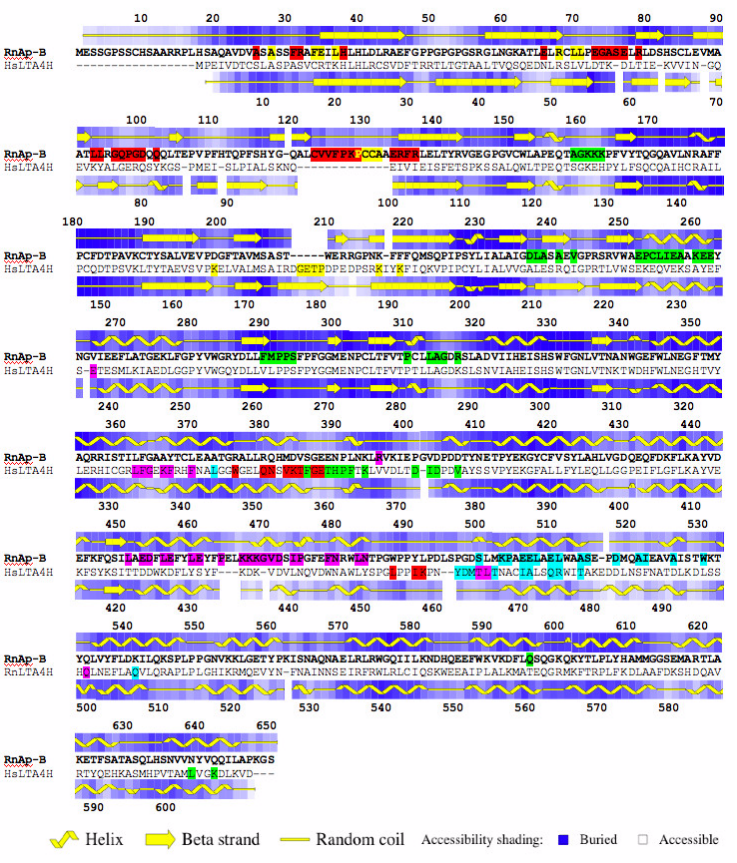
**Comparisons of the primary and secondary structures of rat Ap-B and human LTA_4_H**. Primary structure alignments of rat Ap-B (RnAp-B) and human LTA_4_H (HsLTA4H) are compared to secondary structures deduced from the Ap-B model (upper line) and from the LTA_4_H crystal structure (lower line; PDB accession number:1HS6; [27]) using Procheck software. Residue accessibility computed using the Procheck software are indicated by a color code (see below). Five hydrophobic patches detected in each protein using the Quilt method are also shown in the primary structure of each enzyme. The color code (from larger to smaller patch; see also results section and Figure 8) is the following: red, green, magenta, cyan and yellow, respectively. Dashes typify gaps. Symbols used in the secondary structure schemes are indicated at the bottom of the figure.

The COOH-terminal domain is formed of α-helices arranged into two layers (Fig. 5-A and 5B). The inner layer contains 5 parallel α-helices. Four anti-parallel helices, with perpendicular loops containing short helical segments on top, compose the outer layer. This coil of helices, also found in LTA_4_H structure, looks like Armadillo repeats or HEAT (Hungtington-Elongation-A-subunit-TOR; for review [32]) motif that are suited for protein-protein interactions [33-35]. The super-helical domain of the LTA_4_H primary structure starts from residue 463 and from position 496 for Ap-B (Fig. 5-A, -B; Fig. 6) and it ends with the proteins.

### Structural Analysis

A detailed comparison of both structures highlights differences between Ap-B structural model and the LTA_4_H protein structure. The overall percentages of the different secondary structures differ (Ap-B: α-helix, 42%; β-sheet, 14%; loops, 44% *versus *LTA_4_H: α-helix, 46%; β-sheet, 23%; loops, 31%). In a model deleted from the first 30 NH_2_-terminal residues that are non homologous to LTA_4_H due to the difference in length between both proteins, these percentages can be estimated for Ap-B to: α-helix, 44%; β-sheet, 15%; loops, 41%. As shown in figure 6, this difference in the percentage of β-sheet is mainly due to the presence of gaps in the alignment and to the weak similarity between both proteins in the NH_2_-terminal region (residues 1–133 in Ap-B sequence). Further refinement of the structure could probably slightly modify the loop/β relative percentage but the main features described below would be maintained.

The remaining part of the secondary structural alignment exhibits a high homology (Fig 6). A very small parallel two-stranded β-sheet of three amino acids (LTA_4_H, V^307^TN^309 ^and K_418_SI^420^; Ap-B, V^336^TN^338 ^and Q^448^SI^450^) and a α-helix lying just upstream this β-sheet (LTA_4_H, position 291–300; Ap-B, position 320–329) are conserved between LTA_4_H and Ap-B model (Fig. 6). This helical structure carries the first part of the Zn^2+^-binding motif(HEXXH). In both structures, a large α-helix (LTA_4_H, position 315–334; Ap-B, position 344–363), which contains the third Zn^2+^ligand (E^319^) is located between the two strands of this small β-sheet. This α-helix is followed by a loop (LTA_4_H, position 365–374; Ap-B, position 394–404) that forms and limits one side of the substrate binding pocket.

Then, in both proteins, another α-helix (LTA_4_H, position 382–397; Ap-B, position 412–428), perpendicular to the α-helix containing the HEXXH motif, carries the LTA_4_H Tyr^384^residue (Ap-B, Tyr^414^, 3.25 Å from the Zn^2+ ^ion in the Ap-B model; Fig 5-D) thought to be the proton donor in the catalytic reaction [36]. Lastly, a fourth α-helix (LTA_4_H, position 401–415; Ap-B, position 431–445) takes the primary structure back to the first strand to form the second strand and the small β-sheet. It should be noted that the small parallel two-stranded β-sheet of three amino acids found in Ap-B and LTA_4_H is also conserved in F3 and ePepN structures (data not shown). This small β-sheet structure might be important because it seems to link and to maintain, without too much rigidity considering its size, two important secondary structures containing conserved residues implicated in catalysis and Zn^2+ ^binding.

As described in the LTA_4_H structure [27], a SH3 like motif located in a loop (P-XX-P motif; position 449–462) is also found in the Ap-B model (position 485–499; Fig. 5-C, Fig. 6). However, the Ap-B structural model also exhibits a second proline-rich loop in position 47–57 (Fig. 5-C, Fig. 6). These motifs composed of fairly accessible residues (Fig 6) could constitute putative protein interaction domains.

Analysis of the Ap-B primary structure revealed the presence of a highly conserved RNA binding RNP1 motif (K^416^GYCFVSY^423^; Fig. 6; RiboNucleoProtein 1; [37]) usually encountered in eukaryotic hnRNP and snRNP. The whole RNP motif is, in general, composed of two motifs (RNP2, 6 amino acids; RNP1, 8 amino acids) separated by about 30 amino acid residues.

The Ap-B RNP1 motif (K^416^GYCFVSY^423^) is found in a α-helix upstream the proline-rich loop (position 485–499). RNP1 sequence is masked by this loop and a swinging of this latter structure could render the putative RNA-binding site accessible to its substrate (Fig 5-C; Fig 6). The Tyr^413 ^residue (proton donor) also belongs to this α-helix. No RNP2 consensus sequence is found in the Ap-B primary structure.

**Figure 7:**
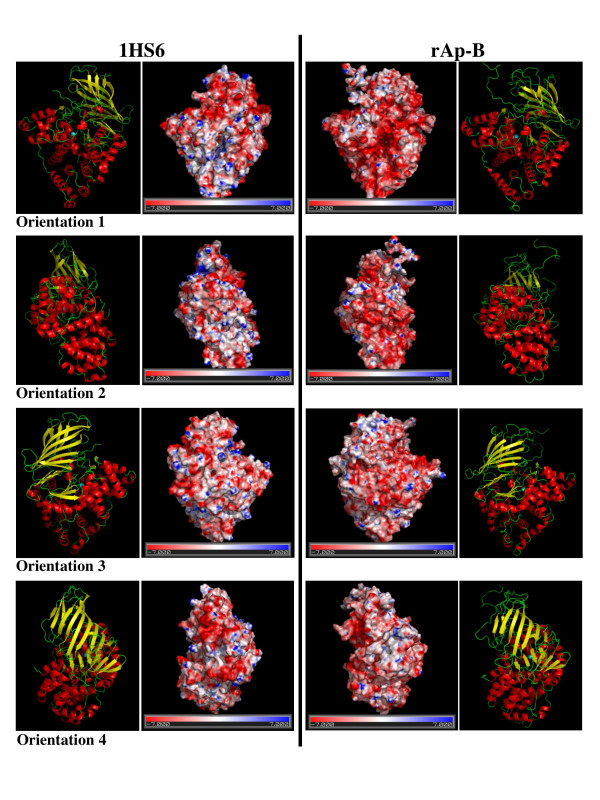
**Electrostatic potential distribution of the LTA_4_H and Ap-B proteins**. Molecular surfaces with electrostatic potentials of both proteins were computed with DELPHI. The potential scale ranges from -7 kT/e (red) to 7 kT/e (blue). Left column, electrostatic potential map displayed on the molecular surface of LTA_4_H (PDB accession number, 1HS6). Right column, electrostatic potential map displayed on the molecular surface of the Ap-B model (rAp-B). Four different orientations (1 to 4; each representing a rotation of about 90°) are represented. The corresponding 3D structure of each protein and orientation are also shown. The figure was created using the molecular graphics software PyMol (PyMol v0.99; [61]).

In order to perform a more precise comparison between the Ap-B model and LTA_4_H, the electrostatic potentials of the molecular surfaces of both proteins were computed. Figure 7 represents electrostatic potential map displayed on the molecular surface of LTA_4_H (PDB accession number, 1HS6; left column) compared to the Ap-B model (rAp-B; right column). Four different orientations with their corresponding 3D ribbon structure are represented showing the 4 different sides of both proteins. The potential scale ranges from -7 kT/e to 7 kT/e (from red to blue). The electrostatic potential distribution between both enzymes is significantly different. rAp-B shows a higher negative potential (Fig 7, Orientation 1, 2 and 3), especially in the catalytic domain. Altogether, the α-helix and the β-sheet domains of Ap-B seem to exhibit a general negative and neutral/positive potentials, respectively. Conversely, the α-helix and the β-sheet domains of LTA_4_H exhibit a general neutral/positive and negative potentials, respectively. Interestingly, orientation 1 in Figure 7 shows, in the centre of the Ap-B molecule corresponding to the active site (substrate binding site), a negative potential versus a neutral/positive potential for LTA_4_H.

**Figure 8:**
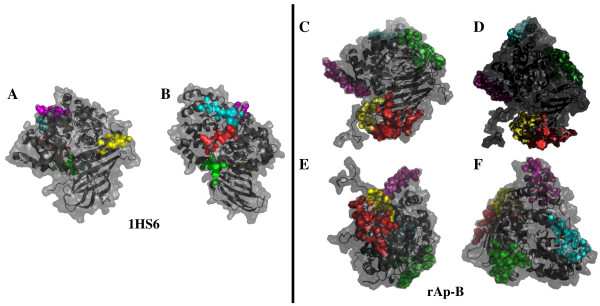
**LTA_4_H and Ap-B hydrophobic patches**. Hydrophobic patches were determined using the Quilt approach. **A-B: **Two different views of LTA_4_H structure (1HS6). The five larger hydrophobic patches of LTA_4_H (298.201 Å^2^, 11 residues, in red; 289.193 Å^2^, 12 residues, in green; 282.972 Å^2^, 10 residues, in magenta; 280.027 Å^2^, 11 residues, in cyan; 262.926 Å^2^, 7 residues, in yellow) are indicated. **C, D, E, F: **Four different views of Ap-B model (rAp-B). The five larger hydrophobic patches of Ap-B (1000.070 Å^2^, 30 residues, in red; 795.016 Å^2^, 31 residues, in green; 645.299 Å^2^, 21 residues, in magenta; 522.096 Å^2^, 15 residues, in cyan; 519.139 Å^2^, 11 residues, in yellow) are also represented. The figure was created using the molecular graphics software PyMol (PyMol v0.99; [61]).

Hydrophobic patches are predicted to be involved in protein-protein interactions. These patches, defined as clusters of neighbouring apolar atoms deemed accessible on a given surface, were investigated on Ap-B and LTA_4_H structures. The total (Ap-B, 26451 Å^2^; LTA_4_H, 23460 Å^2^), hydrophobic (Ap-B, 17550 Å^2^; LTA_4_H, 13629 Å^2^) and hydrophylic (Ap-B, 8893 Å^2^; LTA_4_H, 9831 Å^2^) accessible surfaces of both proteins were determined using the Quilt approach. Ap-B exhibits a slightly more hydrophobic surface (66% of the total surface) than LTA_4_H (58% of the total surface). Concerning the LTA_4_H protein, about 120 small hydrophobic patches were detected. Their surfaces range from 18 to 298.2 Å^2 ^and the clusters contain atoms from 1 to 12 residues. The five larger hydrophobic patches of LTA_4_H (298.201 Å^2^, 11 residues; 289.193 Å^2^, 12 residues; 282.972 Å^2^, 10 residues; 280.027 Å^2^, 11 residues; 262.926 Å^2^, 7 residues) are represented on figure 6 and 8. Some of these patches seem to be associated on the surface of the molecule and might constitute larger hydrophobic surfaces. The LTA_4_H larger hydrophobic patches are mainly located in α-helical domains.

The surface of Ap-B molecule exhibits about 110 hydrophobic patches. Their surfaces range from 4.8 to 1000.070 Å^2 ^and these clusters contain atoms from 5 to 30 residues. The five larger hydrophobic patches of Ap-B (1000.070 Å^2^, 30 residues; 795.016 Å^2^, 31 residues; 645.299 Å^2^, 21 residues; 522.096 Å^2^, 15 residues; 519.139 Å^2^, 11 residues) are also represented on figure 6 and 8. Seven other patches, larger than 300 Å^2 ^(344.229 to 517.249 Å^2^, 9 to 20 residues), were also detected. As for LTA_4_H, several of these patches seem to be associated (Fig. 6 and 8). The association of patch 1 (1000.070 Å^2^, 30 residues, red colour in Fig. 6 and 8) and patch 5 (517.249 Å^2^, 11 residues, yellow colour in Fig. 6 and 8) constitutes a hydrophobic surface of about 1500 Å^2^. If one discards the first largest patch located in an indel region and not yet refined, the second patch is located in the β-sheet domain of the Ap-B structure where the second proline-rich loop (position 47–57; Fig. 5-C) is also found. The three others clusters are situated in α-helical domains.

## Discussion

In this study, we first cloned rat Ap-B CDS into an *E. coli *expression vector. The recombinant rAp-B was produced with a NH_2_-terminal histidine tag and was characterized. The results showed that the expressed protein exhibits the same biochemical properties as its rat counterpart, in terms of its immunoreactivity, kinetic parameters, substrate specificity, NaCl dependence and inhibitor sensitivity. Therefore, this recombinant enzyme was perfectly usable for enzymatic studies.

In order to confirm the functional role of the conserved HEXXHX_18_E motif previously studied in several other enzymes of the M1 family (For review, see [30]), an investigation was conducted by site-directed mutagenesis. As expected, mutagenesis of any of the three zinc ligands of the H^325^EXXH^329^X_18_E^348^ motif in rAp-B sequence or of the critical general base of the peptidase reaction E^326 ^leads to a complete lack of aminopeptidase activity.

In parallel, multiple alignments for the M1 family of aminopeptidases were performed to study the conservation of the residues involved in the exopeptidase activity, these residues constituting eventual targets for further site-directed mutagenesis studies. Our results clearly demonstrate that 3 sub-groups or sub-families constitute the current M1 aminopeptidase family. The main difference between these sub-groups is the presence or the partial loss of a GXMEN motif upstream the Zn^2+^-binding site. The first sub-family is restricted to *Actinobacteria *(loss of Gly, Met, Asn and conservation of Glu). A second sub-family (lack of Gly, Glu, Asn and conservation of Met) is composed of only one member expressed in Vertebrates (Ap-O; [31]). Interestingly, numerous site-directed mutagenesis studies of the GXMEN motif among the M1 family(Aminopeptidase A, Ap-A, [38]; Aminopeptidase N, Ap-N, [39]; Thyrotropine Releasing Hormone Degrading Enzyme, TRH-DE, [40]; LTA_4_H, [41]) suggested that the glutamate and asparagine residues are implicated in the transition state stabilisation. Moreover, these residues, that might interact with the NH_2_-terminus of substrates, are also suspected to be responsible for the aminopeptidase specificity. The Gly residue (an exception is found for TRH-DE, Ala *versus *Gly) seemed crucial for the activity because its replacement with bigger or polar residues abolishes the activity [40]. Lastly, the mutation of the methionine affects both Km and Vm, but no precise role of this residue was proposed [42]. Meanwhile, Ito and collaborators [43] demonstrated that this Met residue is implicated in the recognition of different substrates, explaining the broad specificity of the enzyme. Data about the enzymatic activity of the proteins of the *Actinobacteria *sub-family are not yet available. Diaz-Perales and collaborators demonstrated *in vitro *that Ap-O is an aminopeptidase with specificity for Arg and Asn residues [31]. Further investigation on evolutionary relationships between these proteins and especially on their enzymatic characterization constitute a major goal to grasp the physiological role of these aminopeptidases implicated in fundamental processes of cell metabolism.

In order to investigate the catalytic mechanism of Ap-B, we used molecular modelling. In the present work, we generate a molecular model of the whole rat Ap-B structure based on the crystal structure of the human LTA_4_H [27]. The crystal structures of the Tricorn interacting factor F3 (F3; [44]) and of the aminopeptidase N from *E. coli *(ePepN; [43,45]), two other members of the M1 family that exhibit less than 25 % of identity with LTA_4_H and Ap-B, were considered to be not closely related to Ap-B. Moreover, 4 structural domains exist in F3 [44] and ePepN [43,45]. Interestingly, this fourth domain is inserted just upstream the conserved amino acid region highlighted in the multiple alignment (Fig. 4, position 490). The Ap-B model is quite comparable to the LTA_4_H structure particularly at the level of their catalytic domains. Examination, in both structures, of the distances between Zn^2+ ^ion and the chelating residues His^325^, His^328 ^and Glu^348^, as well as the shape and size of the substrate-binding pocket shows a perfect homology.

The Ap-B structural model was also used to examine *in silico *predicted domains. The Ap-B primary structure contains a high consensual RNA binding RNP1 motif [37]. The whole consensus RNP motif is composed of two motifs RNP2 and RNP1, both are highly conserved and are usually found in simple or multiple copies (1 to 4). RNP domain folds into a compact and hydrophobic αβ structure (βαβ topology, a four anti-parallel β-sheet framed by 2 α-helices perpendicular one to another). RNP1 and RNP2 are found in the two central (underlined) strands, β4-β1–β3-β2. In Ap-B, the absence of RNP2 motif and the fact that RNP1 is not included in a β-sheet could prevent a functional role in RNA binding. Indeed, the presence of this RNP1 motif could be attributed to a vestigial persistence resulting from gene duplications and a loss of nuclear functions in the course of the evolutionary process. Conversely, the presence of a different RNP domain in Ap-B structure could lead to the hypothesis that this protein has gained new interactions via new domains and new structures. RNP1 might be functional and form a particular structure with another motif than RNP2. Indeed, the Ap-B primary structure contains putative nuclear localization sequences and some data supports the hypothesis that this exopeptidase could migrate to the nuclear compartment (unpublished results). Further studies should demonstrate whether Ap-B is also a nuclear protein. In this case, the role of this RNP1 motif will remain to be clarified.

The COOH-terminal domains of LTA_4_H and Ap-B show a structural region similar to the Armadillo repeats or to the HEAT domains. These folds are usually composed of 3 to 36 pairs of anti-parallel α-helices that together form superhelices. The α-helices are commonly 37 to 47 amino acids long in the heat domains and about 40 amino acids long in the armadillo repeats. The known proteins containing these motifs share functions in protein-protein interaction/recognition [33-35]. Although this putative domain in Ap-B and LTA_4_H exhibits shorter α-helices (maximum of 15 residues), the super helical fold present in both structures could lead to variations in the strength of interactions and/or to different interacting proteins.

In addition to the potential HEAT domain, Ap-B exhibits several other features, such as putative SH3 domains, negative electrostatic potential, large hydrophobic patches leading to the hypothesis that this enzyme might interact with different partners in different functions. These characters are generally different from those of LTA_4_H and have been highlighted by the structural model proposed. Differences in the electrostatic potential distribution between Ap-B and LTA_4_H might reflect putative (or different) protein-protein interactions. Ap-B interacts with the outer cytoplasmic membrane of cells and this interaction might occur by protein-protein interactions [4,19]. Ap-B participates, probably with different endopeptidases, to an initial maturation process of neuropeptides and pro-hormones that might involve an endopeptidase/aminopeptidase complex [4,15-18]. Similar roles could be attributed to hydrophobic patches, which delineate putative interacting surface. Ap-B exhibits larger hydrophobic patches than LTA_4_H. On the other hand, the negative electrostatic potential at the level of the catalytic site might explain the difference of enzyme specificity and between their respective substrates: rather negative for LTA_4_H, rather positive for Ap-B. Indeed, a long loop found in both enzymes (Ap-B, K^395^LRVKIEPGVDPDDTY^410^; LTA_4_H, K^364^LVVDLTDIDPDVAY^378^; Fig. 4), delimits and separates the active center from the protein surface. This loop, containing a small α-helix, seems to be involved in substrate specificity as shown by site-directed mutagenesis studies (LTA_4_H, [46]; TRH-DE, [47]; Ap-B, [48]) and structural data (LTA_4_H, [27]). A detailed examination of the corresponding sequences shows that the LTA_4_H sequence is more hydrophobic than the Ap-B sequence, the latter exhibiting a high number of basic amino acids. Moreover, the distribution of acidic residues shows a significant difference in both loops. They are regularly distributed in the LTA_4_H loop structure and rather gathered up one end of the loop in Ap-B, the last aspartate residue belonging to the small α-helix pointing towards the active site.

This might explain the affinity and the positioning of the Ap-B substrate. Basic peptidic substrates might be attracted to the protein surface and to the active site by the negative electrostatic potential. Then, substrate could be guided towards the active center by the presence of hydrophobic and basic residues until stronger interactions occurred involving negatively charged aspartate. The presence of the α-helix might bring some precision and stability to the substrate binding mechanism, balancing the flexibility of the loop.

## Conclusion

The structural model proposed is the first attempt for a better understanding at an atomic scale of the Ap-B protein. The Ap-B 3D model, based on the fold of LTA_4_H, yields important additional information that open the way to design new experiments. For instance, electrostatic potentials computed here, by nature, are features that can only be calculated from a 3D structure, they result from the superposition of the contribution of all the charges of the atoms in a given point. Consequently, a simple sequence analysis cannot foreshadow the electrostatic values. The results we described here illustrate clearly the necessity to go further than simply describing the main fold characteristics to detect fine surface properties of the protein. The important differences in these features compared to LTA_4_H show without ambiguity that the nature or the way the partners interact with Ap-B, the environment impact, would be different from LTA_4_H. Although the complete range of physiological functions of Ap-B remain to be discovered, previous data support the hypothesis that Ap-B is involved in a broad spectrum of relevant physiological phenomena. The specific exopeptidase activity of Ap-B participates in the final steps of neuropeptides and hormones processing (for review, see [15-17,49]). Besides, the enzyme has been implicated in inflammation and/or tumor growth [23,24]. These latter roles might be due to the *in vitro *residual capacity of Ap-B to hydrolyze the LTA_4 _into a lipid mediator of inflammation, the LTB_4 _[18]. In the absence of high-resolution crystal structure, as demonstrated for aminopeptidase A [50,51] and numerous other proteins, this model constitutes a powerful tool for investigating the importance and possible roles of conserved residues in Ap-B and M1 aminopeptidase catalytic sites and to gain new insight into their physiological functions.

## Methods

### Construction of the prokaryotic recombinant rAp-B expression vector

The rat Ap-B cDNA was initially cloned into the SrfI-digested pPCR-Script vector (Stratagene, La Jolla, USA) to obtain the prAp-B plasmid [29]. Both prAp-B and the pIVEX2.4 expression vector (Roche Diagnostics, Meylan, France) were digested with NcoI and BamHI restriction enzymes. The resulting Ap-B cDNA fragment was isolated, purified (QIAquick gel extraction kit, Qiagen, Hilden, Germany) and ligated (T4 DNA ligase, New England Biolabs, Hertfordshire, England) into the digested pIVEX2.4 plasmid to obtain the pIVEX2.4-Ap-B recombinant plasmid, which was checked by sequencing (Genome Express facilities, Meylan, France).

### Production of rat Ap-B in E. coli

In order to produce rAp-B, we used a T7 promoter-driven system and a BLi5 E. coli strain [52]. Ten milliliters of LB medium supplemented with 20 μg/mL chloramphenicol and 100 μg/mL ampicillin were inoculated with Bli5 cells harboring the pIVEX2.4-Ap-B recombinant plasmid and incubated overnight at 37°C with agitation. The overnight culture was then diluted to 1:50 with 50 mL of fresh LB medium containing 100 μg.mL^-1 ^ampicillin and was grown under vigorous shaking at 37°C until the OD_600 _reached 0.6. Isopropyl β-D-1-thiogalactoside (IPTG; Sigma-Aldrich, Saint Quentin Fallavier, France) was added to a final concentration of 1 mM and the expression culture was grown at 37°C with agitation for 2 hours. Cells were then harvested by centrifugation at 4,000 × g for 5 min and stored at -80°C until use or processed immediately as described below for Western blotting, purification and enzymatic activity assays.

### Purification of the recombinant His-tagged rat Ap-B

*Purification using Ni-NTA agarose*. pIVEX2.4-Ap-B transformed cell pellets from an expression culture (see above) were resuspended in 5 mL of lysis buffer (5 mM imidazole, 0.5 M NaCl, 20 mM Tris-HCl, pH 8) and sonicated, at 40 Mcycles, 3 times during 1 min at 4°C. The extract was centrifuged at 10,000 × g for 5 min at 4°C to pellet the cellular debris. The supernatant was collected and mixed with Ni-NTA agarose (v/v; Quiagen, Hilden, Germany), which was pre-equilibrated with lysis buffer. The mixture was incubated with gentle mixing for 1 H at 4°C and applied to a minicolumn for gravity flow chromatography. The column was washed 3 times with 3 to 5 mL of washing buffer (40 mM imidazole, 2 M NaCl, 20 mM Tris-Hcl, pH 8). Finally, the His-tagged purified protein was eluted by 4 successive elutions with a buffer composed of 100 mM imidazole, 200 mM NaCl and 20 mM Tris-HCl at pH 8. *Purification using DEAE ion exchange column*. The recovered supernatant (5 mL, see above) was diluted to 30 mL in 50 mM Tris-HCl pH 7.2, 1 mM β-mercaptoethanol. Then the solution was applied to a DEAE Trisacryl Plus M ion exchange column (100 mL bed volume; BioSepra, Villeneuve la Garenne, France) pre-equilibrated in 50 mM Tris-HCl pH 7.2. Proteins were eluted using a continuous gradient from 0 to 100 mM KCl. The resulting active fractions (5 mL) were concentrated and equilibrated in 50 mM Tris-HCl pH 7.2, 1 mM β-mercaptoethanol using a stirred ultrafiltration cell (Amicon, model 8050; Ultrafiltration membrane YM 30; Millipore Corporation, Bedford, MA, USA).

The determination of the protein concentrations in the different steps of the purification procedure was performed using the Bradford method. The concentration of the purified Ap-B was calculated using its molar absorbance coefficient (105.530 at 280 nm).

### Western blotting

BLi5 or pIVEX2.4-Ap-B transformed cells were resuspended in PBS supplemented with 2 mg/mL lysozyme. Lysis was completed by 15 passages through a 25-gauge needle and 3 sonication steps of 1 min at 40 Mcycles at 4°C. The protein extracts were centrifuged at 4,000 × g during 5 min at 4°C. The supernatants containing the soluble intracellular proteins were retained. Aliquots of protein extracts or purified proteins were run under denaturing conditions on an 8% polyacrylamide gel (SDS-PAGE). Then, they were transferred to a nitrocellulose membrane (0.45 μm, Schleicher & Schuell, Dassel, Germany) using a semi-dry blotting apparatus (Hoefer scientific instruments, San Francisco, USA). The rat Ap-B was detected with a specific anti-Ap-B polyclonal serum [4] at a dilution of 1:2000. Antigen-antibody complexes were visualized using a goat alkaline phosphatase coupled secondary antibody (Sigma-Aldrich, Saint Quentin Fallavier, France) and an NBT-BCIP mixture (Sigma-Aldrich, Saint Quentin Fallavier, France). SDS-PAGE gels were stained with silver salts [53] to visualize the proteins.

### Site-directed mutagenesis

The pIVEX2.4-Ap-B recombinant expression vector was used for site-directed mutagenesis and mutants were generated with the QuickChange^® ^Multi Site-Directed Mutagenesis kit according to the manufacturer specifications (Stratagene Europe, Amsterdam, Netherlands). This system allows randomizing the targeted amino acids using oligonucleotides containing degenerate codons (see below). All the primers used for mutagenesis were 5'-phosphorylated. A single oligonucleotide per site was used in each experiment. The targeted amino acids and their corresponding mutagenic primers were the following: H325, 5'-TGTGGGAGATCTCATRGATGATGACGTCGG-3' for the H325Y and H325H mutants, 5'- TGTGGGAGATCTCGAAGATGATGACGTCGG-3' for the H325F mutant, 5'- TGTGGGAGATCTCGGCGATGATGACGTCGG-3' for the H325A mutant; E326, 5'- ACTGTGGGAGATSTTGTGGATGATGACGTC-3' for the E326Q and E326E mutants, 5'- ACTGTGGGAGATCKCGTGGATGATGACGTC-3' for the E326A and E326E mutants, 5'- ACTGTGGGAGATWTCGTGGATGATGACGTC-3' for the E326D and E326E mutants, 5'- ACTGTGGGAGATGTAGTGGATGATGACGTC-3' for the E326H mutant; H329, 5'-TTCCCAAACCAACTATRGGAGATCTCGTGG-3' for H329Y and H329H mutants, 5'-TTCCCAAACCAACTGAAGGAGATCTCGTGG-3' for H329F mutant, 5'-TTCCCAAACCAACTGGCGGAGATCTCGTGG-3' for H329A mutant; E348, 5'-CATGGTGAAGCCMTCATTGAGCCAGAATTC-3' for the E348D and E348E mutants, 5'-CATGGTGAAGCCCKCATTGAGCCAGAATTC-3' for the E348A and E348E mutants, 5'-CATGGTGAAGCCCTSATTGAGCCAGAATTC-3' for the E348Q and E348E mutants, 5'-CATGGTGAAGCCGTGATTGAGCCAGAATTC-3' for the E348H mutant. Mutants such as H325H, E326E, H329H and E348E are conservative mutants. The mutagenic codon was underlined in the oligonucleotide sequence. The code for degenerate codons was: R = A, G; S = C, G; K = G, T; W = A, T; M = A, C. Generated mutants were identified by direct sequencing on both strands using the dideoxy chain-termination procedure (Genome Express facilities, Meylan, France).

### Enzyme activity assays

Rat Ap-B activity was determined using the L-Arg- and L-Lys-β-naphthylamide (L-Arg-β-NA, L-Lys-β-NA; Sigma-Aldrich; Saint Quentin Fallavier, France) substrates and a specific inhibitor, arphamenine B (Sigma-Aldrich; Saint Quentin Fallavier, France) as previously described in [4]. Briefly, protein extracts were pre-incubated with or without 1 μM arphamenine B for 15 min at 37°C in assay buffer (50 mM Tris-HCl pH 7.4) prior to incubation at 37°C for 60 min in the assay buffer containing 0.2 mM L-Arg-β-NA. Hydrolysis was interrupted by the addition of 0.3 mL of freshly prepared color reagent (Fast Garnet GBC salt; Sigma-Aldrich; Saint Quentin Fallavier, France). The absorbance was read at 535 nm using a spectrophotometer. The percentage of rAp-B activity, which is equivalent to the percentage of inhibition, was measured by comparison with values obtained without inhibitor. Although no other peptidase activities were detectable in purified or semi-purified proteins, activity assays with wild type His-rAp-B enzyme were performed as described above.

### Sequence analysis

Version 2.2.10 of the BLASTP program [54] was used, with default parameters (BLOSUM62 matrix), to identify members of the M1 family of metalloexopeptidases. The Uniprot database (Release 29.0) was queried with the rat Ap-B protein sequence (accession no U61696; [18]) and 512 hits were observed. Among these hits, 500 amino acid sequences exhibiting an E value between 0 and 1.e^-8 ^were selected. These 500 protein sequences were aligned using CLUSTALW version 1.0; [55]. The alignment was refined manually to minimize the number of gaps. Sequences, which exhibited obvious mistranslations resulting from sequencing errors, were deleted from the alignment. The complete listing of the sequence descriptions and the initial and final multiple alignments are available upon request. The PATTERNp program [56] was used to define the consensus amino acid signatures of the M1 family.

### Molecular Modelling

Homology models were constructed as described in [57,58]. Briefly, models were constructed with Modeller package 6.2 version using the 3D structure of the leukotriene A4 hydrolase in complex with Zinc ion and bestatin as a template [27]. In the template structure, the bestatin molecule is located in a tunnel-like cavity. In the model, we do not consider the bestatin molecule for two reasons: i) further theoretical parameters are required for optimizing the whole complexed structure and ii) leaving the cavity free permits a comparison of the difference or similarities in the physico-chemical features of the tunnel in both structures.

The alignment used is described in Figures 4 and 6. Among the 100 models constructed that mainly differ in the indel regions, the 3D model with the optimal objective function was selected ("Initial Model"). Side chains were then repositioned with Scwrl3.0 software. Zinc ion was placed in an equivalent position as observed in the crystal structure of LTA_4_H. Structure refinement was performed with energy minimization *in vacuum *with NAMD package using Charmm force field parameters. A 12 Å cutoff for coulombic interactions was used coupled with 8 Å switching distance. The minimization process was performed in two steps: i) all the atoms of the protein were fixed while the Zn ion was free to move then ii) all the side chains and the Zn ion were free to reorient but the backbone was maintained fixed. The minimization procedure was performed until a gradient tolerance of 10^-6 ^or less was reached. We did not perform any further minimisations freeing all the atoms because of the absence of a proper explicit environment and also for best maintaining the size of the bestatin pocket. The geometric quality of the structure was assessed with Procheck. The quality of the model was tested using 3D-evaluation tools available on the meta-server bioserv [59]. The model was considered to be good with Verify3D, ProsaII and Eval23D, although the average scores were lower with the two last methods.

### Structural Analysis

The structural analysis of the Ap-B model was performed as described in [57,58]. Pocket cavities in the minimized structures were explored using the CastP server.

Electrostatic potentials were computed with Delphi. The dielectric interior and exterior were respectively fixed to 4 and 80. The computation was performed with a null salt concentration. A probe radius equal to 1.4 Å was chosen. The charges and radii correspond to PARSE parameters. The potential was plotted on the surface computed with the MSMS software. A similar computation was performed on the equivalent domain of the LTA_4_H structure, corresponding to the aligned regions between the two sequences.

Hydrophobic patches on the surface were computed according to the Quilt approach. The results depend on different parameters that can be specified: atomic radii, probe radius, polar expansion and point density per atom. The computations were performed with an expansion value of 1.4 Å for the radii of polar atoms (same as the probe radius) as suggested in the original publication. The number of point density per atom was equal to 252. Statistical relevance of patches was evaluated according to the randomization process proposed by the authors. We chose 10 iterations for the assessment.

## Abbreviations

**Ap-B: **aminopeptidase B; **rAp-B: **rat aminopeptidase B; **His-rAp-B: **His-tagged rat aminopeptidase B; **LTA**_4_**H: **leukotriène A_4 _hydrolase; **LTA**_4_**: **leukotriène A_4_; **LTB**_4_**: **leukotriène B_4_; **His-tag**: histidine tag; **PMSF: **Phenylmethylsulfonyl Fluoride; **NEM: **N-ethylmaleimide; **Ap-O: **aminopeptidase O; **TRH-DE: **thyrotropin-releasing hormone degrading enzyme, **HEAT domain: **Hungtington-Elongation-A-subunit-TOR; **RNP**: ribonucleoprotein; **F3: **Tricorn interacting factor F3; **ePepN: **aminopeptidase N from *E. coli*.

## Authors' contributions

VLP, MSC, CGD, MCB and TF participated in the construction of the Ap-B expression vector. VLP, MSC, CGD and CH carried out the production of the recombinant enzyme, participated in the purification of the enzyme and in its biochemical characterization. VLP and TF carried out the multiple alignments and all authors helped to its final version. VLP, MSC, CGD, CH and TF participated in site-directed mutagenesis and nucleotide sequences analysis. CE carried out molecular modelling. VLP, MSC, PN and CE were involved in the structural analysis. MSC, PN and TF conceived the study and together with VLP were responsible for study design and coordination. TF was project manager of the ARC 5868 project under which this study was performed. VLP, MSC, CE and TF drafted the manuscript and MCB and PN critically read the manuscript. All authors have read and participated in the revision of the text and figures and approved the final submitted version.

## Supplementary Material

Additional file 1 Structural model of rAp-B. The data provided allow visualizing the 3D structural model of rAp-B. Click here for file
